# Lung Surfactant Decreases Biochemical Alterations and Oxidative Stress Induced by a Sub-Toxic Concentration of Carbon Nanoparticles in Alveolar Epithelial and Microglial Cells

**DOI:** 10.3390/ijms22052694

**Published:** 2021-03-07

**Authors:** Giuseppe Caruso, Claudia G. Fresta, Angelita Costantino, Giacomo Lazzarino, Angela M. Amorini, Giuseppe Lazzarino, Barbara Tavazzi, Susan M. Lunte, Prajnaparamita Dhar, Massimo Gulisano, Filippo Caraci

**Affiliations:** 1 Department of Drug and Health Sciences, University of Catania, 95125 Catania, Italy; angelita25costantino@gmail.com (A.C.); m.gulisano@unict.it (M.G.); carafil@hotmail.com (F.C.); 2 Department of Biomedical and Biotechnological Sciences (BIOMETEC), University of Catania, 95125 Catania, Italy; forclaudiafresta@gmail.com (C.G.F.); amorini@unict.it (A.M.A.); lazzarig@unict.it (G.L.); 3 Interuniversity Consortium for Biotechnology, Area di Ricerca, Padriciano, 34149 Trieste, Italy; 4 UniCamillus-Saint Camillus International University of Health Sciences, 00131 Rome, Italy; giacomo.lazzarino@unicamillus.org; 5 Department of Basic Biotechnological Sciences, Intensive and Perioperative Clinics, Catholic University of the Sacred Heart of Rome, 00168 Rome, Italy; barbara.tavazzi@unicatt.it; 6 Fondazione Policlinico Universitario A. Gemelli IRCCS, 00168 Rome, Italy; 7 Ralph N. Adams Institute for Bioanalytical Chemistry, University of Kansas, Lawrence, KS 66047-1620, USA; slunte@ku.edu; 8 Department of Pharmaceutical Chemistry, University of Kansas, Lawrence, KS 66047-1620, USA; prajnadhar@ku.edu; 9 Department of Chemistry, University of Kansas, Lawrence, KS 66047-1620, USA; 10 Department of Chemical and Petroleum Engineering, University of Kansas, Lawrence, KS 66045-7576, USA; 11 Molecular Preclinical and Translational Imaging Research Centre-IMPRonTE, University of Catania, 95125 Catania, Italy; 12 Oasi Research Institute-IRCCS, 94018 Troina (EN), Italy

**Keywords:** toxicology, carbon nanoparticles, reactive oxygen species (ROS), energy metabolism, mitochondrial dysfunction, alveolar epithelial cells, microglia

## Abstract

Carbon-based nanomaterials are nowadays attracting lots of attention, in particular in the biomedical field, where they find a wide spectrum of applications, including, just to name a few, the drug delivery to specific tumor cells and the improvement of non-invasive imaging methods. Nanoparticles inhaled during breathing accumulate in the lung alveoli, where they interact and are covered with lung surfactants. We recently demonstrated that an apparently non-toxic concentration of engineered carbon nanodiamonds (ECNs) is able to induce oxidative/nitrosative stress, imbalance of energy metabolism, and mitochondrial dysfunction in microglial and alveolar basal epithelial cells. Therefore, the complete understanding of their “real” biosafety, along with their possible combination with other molecules mimicking the in vivo milieu, possibly allowing the modulation of their side effects becomes of utmost importance. Based on the above, the focus of the present work was to investigate whether the cellular alterations induced by an apparently non-toxic concentration of ECNs could be counteracted by their incorporation into a synthetic lung surfactant (DPPC:POPG in 7:3 molar ratio). By using two different cell lines (alveolar (A549) and microglial (BV-2)), we were able to show that the presence of lung surfactant decreased the production of ECNs-induced nitric oxide, total reactive oxygen species, and malondialdehyde, as well as counteracted reduced glutathione depletion (A549 cells only), ameliorated cell energy status (ATP and total pool of nicotinic coenzymes), and improved mitochondrial phosphorylating capacity. Overall, our results on alveolar basal epithelial and microglial cell lines clearly depict the benefits coming from the incorporation of carbon nanoparticles into a lung surfactant (mimicking its in vivo lipid composition), creating the basis for the investigation of this combination in vivo.

## 1. Introduction

The ideas and concepts behind nanotechnology, initially defined as the process in which scientists would be able to manipulate and control individual atoms and molecules, were first introduced by Nobel laureate Richard Feyman during his famous lecture entitled “There’s Plenty of Room at the Bottom” in the late 1950s [1]. Over a decade later, Professor Norio Taniguchi coined the term “nanotechnology” as the science conducted at the nanoscale, ranging from 1 to 100 nanometers (nm) [2].

Among the different types of available nanomaterials, engineered nanoparticles (ENPs), because of their physicochemical and biological properties, including strength, flexibility, performance, and durability [3], have attracted thoughtful attention in the biomedical field. EPNs are currently studied to be used for targeted drug delivery to tumors [4,5,6], to break up clusters of bacteria, enhancing bacterial killing [7], for stimulation of immune responses [8], for improvement of non-invasive imaging methods [9], and for scavenging of reactive oxygen species (ROS) [10]. The increased specific surface area allows these particles to transport therapeutic agents, biomarkers, and ligands more efficiently, as compared to the conventional drug delivery systems, having the potential to radically change the method of administering drugs [11].

Considering the carbon-based nanoparticles, non-toxic diamond nanoparticles (engineered carbon nanodiamonds, ECNs) represent very promising drug delivery vehicles allowing greater treatment sensitivity and more patient-specific dosages [12,13,14,15,16]. Unfortunately, there is a “but”; in fact, despite their enormous potential in terms of applications, some aspects concerning the possible toxicity and side effects of nanoparticles, the complexity in the management of drug administration (nanodiamond/drug interface), or the difficulty of purifying the nanodiamonds with a high yield have not yet been clarified [11].

Nanoparticles and related agglomerates with a size ranging from 10 to 200 nm could be inhaled during breathing, accumulating in the lung alveoli and then interacting with the so-called lung surfactants (LSs), a complex mixture of essential molecules, such as lipids, proteins, and carbohydrates [17]. LSs, in order to prevent the collapse of alveoli, reduce the surface tension at the air–water interface in these structures at end-expiration [18] and also represent the first line against several external particles [19].

Since most probably the inhaled ECNs, and in general ENPs, will not be confined in the respiratory tract, reaching relevant human tissues different than lungs, the investigation to weigh their beneficial and damaging effects in different experimental models appears certainly relevant. In this regard, a recent application of ECNs in the biomedical field is represented by their use as a drug delivery system for intracranial tumor treatment [16], as well as in neurodegenerative disorders such as Alzheimer’s disease (AD) [20].

It is with the above in mind that several studies evaluated carbon-based nanomaterials in terms of biosafety, toxicity, and/or possible side effects both in vitro and in vivo [21]. In particular, the characterization of the potential toxicity of ECNs revealed heterogeneous cytotoxic effects, including the generation of ROS, DNA damage, lysosomal damage, and mitochondrial dysfunction, eventually culminating in cell death by apoptosis or necrosis [22]. We recently evaluated in alveolar basal epithelial (A549) and brain microglial (BV-2) cells, whether an apparently non-toxic concentration of ECNs was able to cause cellular biochemical alterations. Under these experimental sub-toxic conditions, we found that ECNs-challenged cells underwent an increase in nitric oxide (NO) and ROS production, as well as a deregulation of mitochondrial functions and energetic metabolism [23]. In the aforementioned study, A549 cells (alveolar basal epithelial phenotype) were selected because the lung represents a primary site of nanoparticle retention after inspiration [17] and are also a preference model to study toxicity linked to ROS overproduction [24,25,26], while BV-2 cells (microglial phenotype) were selected as a valid model system alternative to primary microglia cultures [27,28], highly responsive to inflammatory stimuli and trophic factors [29].

The focus of the present work was to investigate whether the biochemical alterations of cell metabolism and functions, induced by an apparently non-toxic concentration of ECNs, could be counteracted/modulated by their incorporation into the LS-like model mixture represented by DPPC:POPG in a 7:3 molar ratio. In particular, the effects of sub-toxic, non-proliferating ECNs, alone or incorporated into DPPC:POPG(7:3), on NO and total ROS production, metabolites related to energy metabolism, mitochondrial phosphorylating capacity, total pool of nicotinic coenzymes, and antioxidant defenses in A549 and BV-2 cells was investigated.

## 2. Results

### 2.1. The Presence of LS Does Not Affect Cell Death and Proliferation

**Figure 1:**
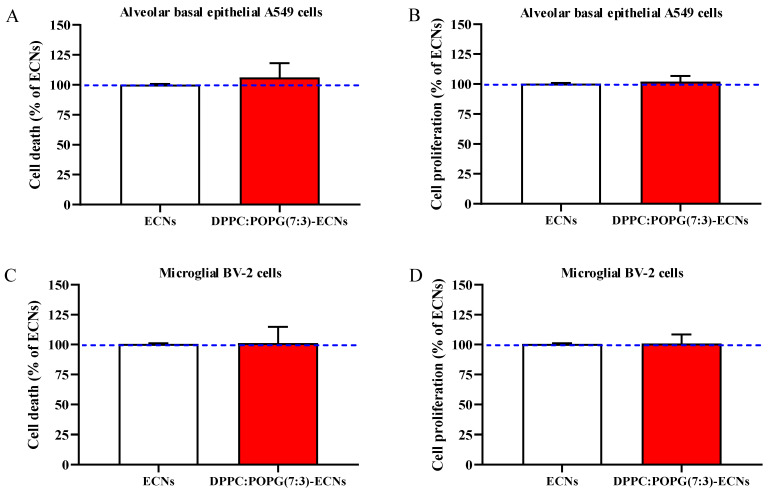
Cell viability and proliferation of A549 (**A**,**B**) and BV-2 (**C**,**D**) cells following 24 h of engineered carbon nanodiamonds (ECNs) (2 µg/mL) challenge without and with the lung surfactant (LS) DPPC:POPG(7:3) (indicated as DPPC:POPG(7:3)-ECNs). DPPC = dipalmitoyl phosphatidylcholine; POPG = 1-palmitoyl-2-oleoyl-sn-glycero-3-phospho-(1′-rac-glycerol). Data are the mean of three independent experiments and are expressed as the percent variation with respect to the cell viability or proliferation recorded in ECNs-treated cells. Standard deviations are represented by vertical bars.

The first aim of the present study was to evaluate potential differences in cell death and proliferation occurring to A549 and BV-2 cells challenged with ECNs 2 µg/mL alone or in combination with our surfactant model (DPPC:POPG(7:3)), as a coating lung biomaterial of occasionally inhaled nanoparticles. As clearly showed in Figure 1, no significant differences between the two treatments (24 h) in cell death (A and C) and proliferation (B and D) were observed in both A549 and BV-2 cells.

### 2.2. The Presence of LS Decreases the Production of ROS Induced by a Sub-Toxic Concentration of ECNs

**Figure 2:**
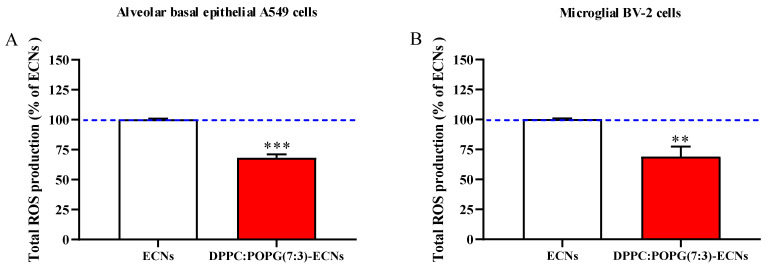
Total reactive oxygen species (ROS) production of A549 (**A**) and BV-2 (**B**) cells following 24 h of ECNs (2 µg/mL) challenge without and with the LS DPPC:POPG(7:3) (indicated as DPPC:POPG(7:3)-ECNs). ECNs = engineered carbon nanodiamonds; DPPC = dipalmitoyl phosphatidylcholine; POPG = 1-palmitoyl-2-oleoyl-sn-glycero-3-phospho-(1′-rac-glycerol). Data are the mean of three independent experiments and are expressed as the percent variation with respect to the total ROS production recorded in ECNs-treated cells. Standard deviations are represented by vertical bars. ** Significantly different from ECNs-treated cells, *p* < 0.01; *** significantly different from ECNs-treated cells, *p* < 0.001.

In a different set of experiments, we investigated whether the presence of LS is able to decrease the well-known ability of 2 µg/mL ECNs, to induce ROS overproduction. Results demonstrate that the presence of the LS (DPPC:POPG(7:3)/ECNs) significantly decreased the amount of total intracellular ROS in both A549 (Figure 2A) and BV-2 (Figure 2B) cells (−32%, *p* < 0.001 and −31%, *p* < 0.01, respectively, compared to ECNs).

### 2.3. LS Ameliorates Energy State and Mitochondrial Activity of Alveolar Epithelial and Microglial Cells Challenged with ECNs

**Figure 3:**
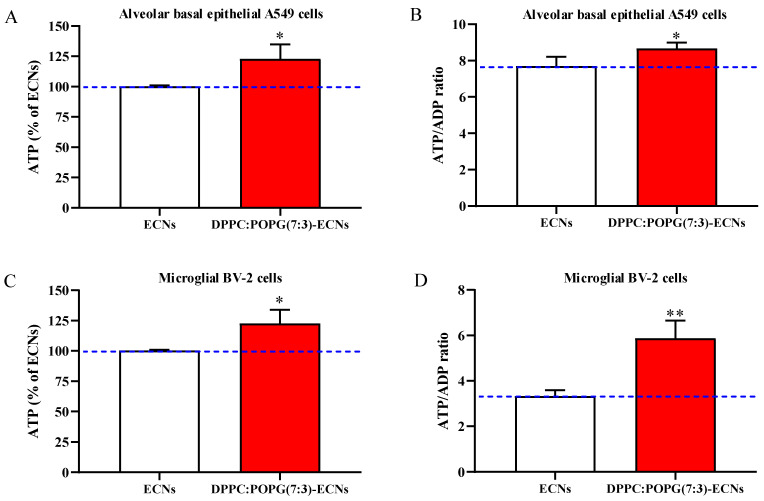
Values of adenosine triphosphate (ATP) and mitochondrial phosphorylating capacity (ATP/adenosine diphosphate (ADP) ratio) of A549 (**A**,**B**) and BV-2 (**C**,**D**) cells following 24 h of ECNs (2 µg/mL) challenge without and with the LS DPPC:POPG(7:3) (indicated as DPPC:POPG(7:3)-ECNs). ECNs = engineered carbon nanodiamonds; DPPC = dipalmitoyl phosphatidylcholine; POPG = 1-palmitoyl-2-oleoyl-sn-glycero-3-phospho-(1′-rac-glycerol). In the case of (**A**,**C**), data are the mean of three independent experiments and are expressed as the percent variation, with respect to the quantity of ATP recorded in ECNs-treated cells. Standard deviations are represented by vertical bars. * Significantly different from ECNs-treated cells, *p* < 0.05; ** significantly different from ECNs-treated cells, *p* < 0.01.

In our previous experiments we demonstrated that both energy state and mitochondrial activity, evaluated in terms of ATP (the primary source for all the energy-requiring cells processes) [30] and ATP/ADP ratio (a reliable index of the mitochondrial phosphorylating capacity) [31], of A549 and BV-2 cells are negatively affected by 2 µg/mL ECNs. In these new experiments, the addition of LS to 2 µg/mL ECNs produced an increase in the intracellular ATP concentration that was comparable in both cell lines (Figure 3A,C), (*p* < 0.05, compared to ECNs).

In accordance with this effect on ATP levels, the ATP/ADP ratio significantly increased in A549 cells treated with DPPC:POPG(7:3)/ECNs, compared to the values of cells treated with ECNs only (+12%, *p* < 0.05) (Figure 3B). In the BV-2 line, the amelioration of the ATP/ADP ratio was more remarkable when cells were challenged with DPPC:POPG(7:3)/ECNs, as compared to the values recorded in cells treated with ECNs only (+80%, *p* < 0.01) (Figure 3D).

### 2.4. LS Increases the Cellular Pool of Nicotinic Coenzymes

**Figure 4:**
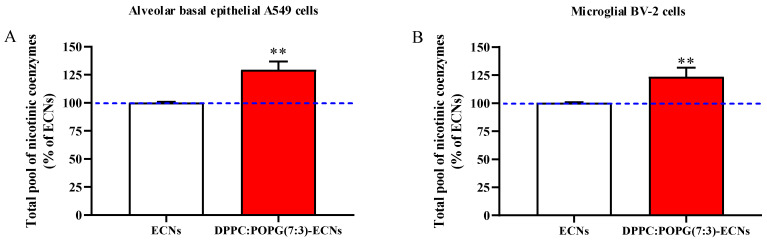
Total pool of nicotinic coenzymes (NAD+ + NADH + NADP+ + NADPH) in A549 (**A**) and BV-2 (**B**) cells following 24 h of ECNs (2 µg/mL) challenge without and with the LS DPPC:POPG(7:3) (indicated as DPPC:POPG(7:3)-ECNs). ECNs = engineered carbon nanodiamonds; DPPC = dipalmitoyl phosphatidylcholine; POPG = 1-palmitoyl-2-oleoyl-sn-glycero-3-phospho-(1′-rac-glycerol); NAD^+^ = oxidized nicotinamide adenindinucleotide; NADH = reduced nicotinamideadenindinucleotide; NADP^+^ = oxidized nicotinamideadenindinucleotide phosphate; NADH = reduced nicotinamideadenindinucleotide phosphate. Data are the mean of three independent experiments and are expressed as the percent variation with respect to the total pool of nicotinic coenzymes recorded in ECNs-treated cells. Standard deviations are represented by vertical bars. ** Significantly different from resting, *p* < 0.01.

The ability of LS to counteract the perturbation of energy metabolism was also evaluated by measuring the total pool of cellular nicotinic coenzymes [32]. As shown in Figure 4A,B, the presence of lung surfactant increased in a similar manner the total pool of nicotinic coenzymes (NAD^+^ + NADH + NADP^+^ + NADPH) in both A549 and BV-2 cells (+29% and + 22%, respectively; *p* < 0.01 compared to cells challenged with 2 μg/mL ECNs only).

### 2.5. LS Protects GSH Levels and Decreases Oxidative/Nitrosative Stress

**Figure 5:**
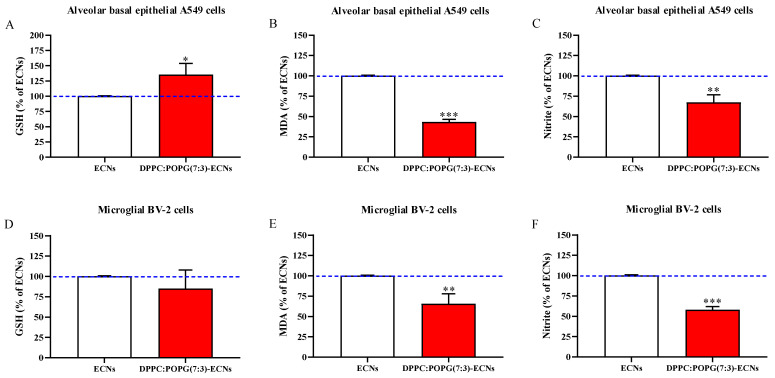
GSH, MDA, and nitrite in A549 (**A**–**C**) and BV-2 (**D**–**F**) cells following 24 h of ECNs (2 µg/mL) challenge without and with the LS DPPC:POPG(7:3) (indicated as DPPC:POPG(7:3)-ECNs). ECNs = engineered carbon nanodiamonds; DPPC = dipalmitoyl phosphatidylcholine; POPG = 1-palmitoyl-2-oleoyl-sn-glycero-3-phospho-(1′-rac-glycerol); GSH = reduced glutathione; MDA = malondialdehyde. Data are the mean of three independent experiments and are expressed as the percent variation with respect to the quantity of GSH, MDA, or nitrite recorded in ECNs-treated cells. Standard deviations are represented by vertical bars. * Significantly different from ECNs-treated cells, *p* < 0.05; ** Significantly different from ECNs-treated cells, *p* < 0.01; *** Significantly different from ECNs-treated cells, *p* < 0.001.

Figure 5 depicts the effects of LS, expressed as the percent variation with respect to ECNs-treated cells, on the levels of GSH, the most important water-soluble low-molecular weight antioxidant protecting free-SH groups of proteins, of malondialdehyde (MDA), a stable end-product of ROS-mediated peroxidation of fatty acids of membrane phospholipids, and of nitrite, a stable end-product of NO metabolism.

As clearly showed in Figure 5A, the treatment with DPPC:POPG(7:3)/ECNs significantly increased intracellular GSH in A549 cells (+45%; *p* < 0.05 compared to ECNs-treated cells), whilst the presence of LS was ineffective in the case of BV-2 cells (Figure 5D). A decrease in oxidative stress in both A549 and BV-2 cells treated with DPPC:POPG(7:3)/ECNs (Figure 5B,E) was evidenced by a significant 60% and 36% diminution, respectively, of intracellular MDA levels (*p* < 0.001 and *p* < 0.01, respectively, compared to ECNs-treated cells). A similar trend was observed when measuring nitrite as an indirect biomarker of NO production. In this case (Figure 5C,F), a significant decrease of −30% (*p* < 0.01) and −42% (*p* < 0.001) was measured in A549 and BV-2 cells, respectively, compared with ECNs-treated cells.

## 3. Discussion

As previously mentioned, the use of carbon-based materials in different fields, such as biology and medicine, is attracting a lot of attention. In particular, carbon nanoparticles have been recently evaluated as an alternative, innovative, and powerful tool for drug delivery and cancer therapy [33] and have also been considered as a potential therapeutic strategy with regards to neurodegenerative diseases [34], including AD [35,36]. Therefore, the evaluation of their safety, toxicity and/or possible side effects in vitro and in vivo, as well as of the combination of nanoparticles with molecules mimicking the in vivo milieu able to counteract their negative effects becomes of utmost importance. Moreover, to date, no results have already been produced on the ability of LSs, representing the first line of defense against any foreign particles [19], to counteract the biochemical alterations and oxidative stress induced by a sub-toxic concentration of ECNs on alveolar epithelial and microglia cells.

We recently demonstrated that an apparently non-toxic ECN concentration leads to oxidative/nitrosative stress, imbalance of energy metabolism, and mitochondrial dysfunction in alveolar basal epithelial (A549) and brain microglial (BV-2) cells. The experiments described in the present study were purposely designed to determine, in the two above-mentioned cell lines, whether the incorporation of ECNs in DPPC:POPG(7:3), mimicking the composition of LS, is capable to counteract the negative effects related to the use of this specific carbon nanoparticles.

As clearly shown in Figure 1, we firstly demonstrated that the presence of LS, as a coating lung biomaterial of occasionally inhaled nanoparticles, did not significantly affect cell death and proliferation in A549 or BV-2 cell lines. These findings might be of particular relevance in view of the conflicting literature regarding lung surfactant’s biosafety [37]. In fact, as shown by Kasper et al. in A549 cells, pulmonary surfactant augments cytotoxicity of silica nanoparticles [38]. In a different set of experiments, we showed that the presence of LS was able to significantly decrease the production of ROS induced by ECNs in both cell lines (Figure 2). This decreased production could be due to direct or indirect activity of LS. On one hand, pulmonary surfactant could directly be exposed and interact with the ROS produced by activated immune cells (e.g., leukocytes and macrophages) in the alveolar space [39], being inactivated but at the same time decreasing the amount of free species [40]; on the other hand, lung surfactants could indirectly decrease ROS, by modulating the expression of specific genes related to reactive species production. Indeed, Crowther et al. showed that pulmonary surfactant protein A is able to inhibit the production of ROS in macrophages by reducing the activity of NADPH oxidase enzyme [41].

Previous findings demonstrated that energy state and mitochondrial activity, evaluated in terms of ATP [30] and ATP/ADP ratio [31], respectively, are negatively affected in A549 and BV-2 cells by the challenge with ECNs. Here we show that, compared to ECNs only, the addition of LS to ECNs (DPPC:POPG(7:3)/ECNs) induced a significant increase in the intracellular ATP concentration in both cell lines (Figure 3A,C), paralleled by an increase in the mitochondrial phosphorylating capacity, evaluated by the ATP/ADP ratio (Figure 3B,D). This clearly depicts the ability of LS to counteract the cellular energy depletion caused by the imbalance in the main mitochondrial function, i.e., the adequate supply of ATP essential to ensure all the energy consuming reactions taking place inside the cells. The ability of LS to ameliorate the ATP/ADP ratio in both cell lines could be of great interest, since it has been shown that under mitochondrial malfunctioning with energy penalty the complex mechanisms related to mitochondrial quality control, involving numerous genes and proteins able to regulate the mitochondrial status [42,43], tend towards fission and mitophagy [44,45], two processes strictly correlated to dysregulated cell metabolism phenomena.

As regards the phosphorylating capacity, it is worth noting that the amelioration of the ATP/ADP ratio was more remarkable in microglial cells than in alveolar epithelial cells. This can be explained, at least in part, by the very different phenotypes (microglial and epithelial) considered in this study. In fact, to date, microglia are considered among the most versatile cells in the body, having the ability to morphologically and functionally adapt to their constantly changing surrounding environment. Of note, even in a resting state, the processes of microglia are highly dynamic and perpetually scan the central nervous system [46], with a very efficient mitochondrial oxidative phosphorylation and production of ATP [47].

The incorporation of ECNS into LS increased the cellular pool of nicotinic coenzymes (NAD^+^, NADH, NADP^+^, and NADPH) in both cell lines, compared to the values found in cells challenged with ECNs only (Figure 4). This avoided both the decrement of the reducing equivalents supply needed for the correct functioning of the mitochondrial electron transfer chain in A549 and BV-2 cells, and the diminution in the efficiency of the nicotinic coenzyme-dependent oxido-reductive reactions [23]. These positive activities of LS could be of relevance in conditions characterized by energy failure, such as myocardial ischemia [48], brain injuries [32,49], and AD [50], all pathological conditions for which the therapeutic use of nanoparticles has been recently considered [51,52,53].

According to the present results obtained in the last set of experiments (Figure 5), the treatment with DPPC:POPG(7:3)/ECNs significantly increased intracellular GSH, the most important water-soluble low-molecular-weight antioxidant protecting free-SH groups of proteins [54], but only in the case of A549 cells, whilst the same treatment was ineffective in the case of BV-2 cells. The different effects observed in the two cell lines could be the result of the higher reactivity of microglial cells compared to epithelial cells; in fact, it has been reported by Rojo et al. that the loss of GSH in microglial cells could be due to its participation in redox or conjugation reactions, or alternatively, to its export from the cell. Additionally, during ROS detoxification, GSH levels decreased because of its oxidation to GSSG [55]. Very importantly, a significant decrease in oxidative/nitrosative stress, evaluated by measuring intracellular MDA and nitrite levels, was observed in both cell lines, when LS end-capped ECNs (Figure 5). The use of LSs could then be take into consideration in the case of the administration of nanoparticles that, despite their therapeutic potential, have not been employed due to their association with the induction of oxidative/nitrosative stress [56,57]. Lastly, since carbon-based nanomaterials, and in particular ECNs, have been considered for drug-delivery systems [58] or as anticancer agents [59], the new information obtained in our experiments could be of significant benefit for the further development of these highly relevant applications.

In summary, our results indicate that the incorporation of ECNs into LS (1) does not affect cell death and proliferation; (2) decreases the intracellular production of ROS induced by ECNs; (3) improves energy state and mitochondrial activity; (4) increases the intracellular pool of nicotinic coenzymes; and (5) increases GSH levels (in A549 cells only) and decreases oxidative/nitrosative stress phenomena.

## 4. Materials and Methods

### 4.1. Materials and Reagents

All materials and reagents were of analytical grade and purchased from Sigma-Aldrich (St. Louis, MO, USA) or Thermo Fisher Scientific (Waltham, MA, USA), unless differently specified. BV-2 (microglial) cells (ICLC ATL03001) were purchased from Interlab Cell Line Collection (ICLC, Genova, Italy), while A549 (alveolar basal epithelial) cells (ATCC^®^ CCL-185™), RPMI-1640 medium, fetal bovine serum (FBS), trypsin-EDTA solution, and penicillin/streptomycin solution were purchased from American Type Culture Collection (ATCC, Manassas, VA, USA). High-performance liquid chromatography (HPLC)-grade methanol, far-UV acetonitrile, and HPLC-grade chloroform were supplied by J. T. Baker Inc. (Phillipsburgh, NJ, USA). Polyethersulfone membrane (3K) was supplied by VWR International (West Chester, PA, USA). C-Chip disposable hemocytometer, used for cell counting, was purchased from Bulldog Bio, Inc. (Portsmouth, NH, USA). Curing agent and sylgard 184 polydimethylsiloxane prepolymer, used for the preparation of microchips, were purchased from Ellsworth Adhesives (Germantown, WI, USA). Organic (chloroform) mixtures of the phospholipids, 1-palmitoyl-2-oleoyl-sn-glyc-ero-3-phospho-(1′-rac-glycerol) (POPG, 10 mg/mL), and dipalmitoyl phosphatidylcholine (DPPC, 25 mg/mL) were obtained from Avanti Polar Lipids Inc. (Alabaster, AL, USA). ECNs used in this research were obtained from Microdiamant (Lengwil, Switzerland). Nitrogen tanks for drying were supplied by Matheson Tri-Gas Inc. (Basking Ridge, NJ, USA). All water used in our study was Ultrapure (18.3 MΩ cm) (Milli-Q Synthesis A10, Millipore, Burlington, MA, USA).

### 4.2. Preparation of Nanoparticle Suspensions

Each solution of DPPC:POPG (7:3 molar ratio) was prepared by using HPLC-grade chloroform. This specific molar ratio was selected as a model mixture to emulate the composition of other synthetic LSs [60,61]. After a drying step (nitrogen), the lipid mixtures were kept under vacuum, overnight. Taking into consideration the transition phase temperature of DPPC (41 °C), PBS at pH 7.4 was used to re-suspend the dried lipids (10 mg/mL) in a Precision water bath system at 45 °C. PBS suspensions of ECNs were sonicated for 2 h to prevent any aggregation of the nanoparticle mixture and then used either alone or in combination with LS at the concentration of 2 µg/mL. Data on the full characteristics of nanoparticles have been fully described in previous studies [23,62].

### 4.3. *Propagation* and *Maintenance* of *Cells*

A549 and BV-2 cells were cultured in RPMI-1640 containing 10% (*v*/*v*) FBS, streptomycin (0.3 mg mL^–1^), and penicillin (50 IU mL^–1^) by using 75 cm^2^ polystyrene culture flasks (5 × 10^6^ cells). Cells were maintained in a humidified environment (37 °C, 5% CO_2_), and passaged every 3–5 days, in order to avoid cell overgrowth.

### 4.4. Analysis of Cell Viability and Proliferation

On the day of the experiment, A549 and BV-2 cells were harvested, counted, and plated in 48-well plates (15 × 10^4^ cells/well). After 2 h, cells were treated with ECNs or DPPC:POPG(7:3)/ECNs and incubated for 24 h, in a humidified environment (37 °C, 5% CO_2_). At this time point, the effects of the two different experimental conditions on cell viability and cells proliferation were measured through the MTT method [63,64] and the Cell Proliferation Kit II (XTT), following the manufacturer’s instructions.

### 4.5. Intracellular ROS Levels Determination

After 24 h incubation with the treatments, the intracellular ROS levels in A549 and BV-2 cells were determined by using microchip electrophoresis with laser-induced fluorescence (ME-LIF) and 2′,7′-dichlorodihydrofluorescein diacetate (H_2_DCFDA) probe. After removal of the suspending medium, cells were washed twice with PBS and then incubated with phenol red-free RPMI-1640 containing H_2_DCFDA probe (final concentration of 10 μM). Stock solution of H_2_DCFDA probe (10 mM) was freshly prepared before each experiment. The probe was allowed to react for 60 min in a humidified environment (37 °C, 5% CO_2_), at the end of which the medium containing the probe was removed; cells were washed with PBS and harvested, using a trypsin-EDTA solution (2.5 mL; 0.25% Trypsin/0.53 mM EDTA in Hanks Balanced Salt Solution without calcium or magnesium). An aliquot (100 µL) of the cell suspension was always removed for cell counting before to carry out a centrifugation step (125× *g* for 5 min at 4 °C). The resulting cell pellet was washed, using PBS, and lysed by adding 50 µL of pure ethanol. The obtained solution was filtered by using centrifuge tubes equipped with 3 kDa molecular weight cut-off filters (18.690× *g* for 10 min at 4 °C) [65]. To prepare the sample into a form ready for analysis, 10 µL of the filtered solution was added to 90 µL of running buffer (10 mM boric acid and 7.5 mM SDS at pH 9.2) and then analyzed with the microfluidic device [66].

The fabrication of hybrid polydimethylsiloxane(PDMS)-glass microfluidic devices used to perform the ME-LIF experiments has been described previously in details [66,67,68]. Prior to each cell lysate analysis, a NaOH (0.1 M for 5 min) and a running buffer (10 mM boric acid, 7.5 mM SDS at pH 9.2 for 5 min) solutions were used to flush the PDMS-glass device. Each separation was carried out by using a 30 kV high-voltage power supply (Ultravolt, Ronkonkoma, NY, USA). Then, +2400 V was applied to the running buffer reservoir, while +2200 V was used in the case of sampling reservoir. A 1 s gated injection was set for the introduction of each sample into the separation channel of the microfluidic device. The presence of any residual sample on the channels between two runs was prevented by flushing the separation channel (~60 s) with the running buffer. The same technologies and software previously described [69] were employed to achieve the needed excitation as well as detection, data acquisition, and data analysis.

### 4.6. Analysis of Metabolites

The analysis of intracellular metabolites in deproteinized samples of A549 and BV-2 cells was performed by HPLC. To this purpose, at the end of 24 h incubation with ECNs or DPPC:POPG(7:3)/ECNs, cells were pelleted (at 4 °C) and washed twice with ice-cold PBS (pH 7.4). Immediately after the second washing step, cells were deproteinized according to the organic solvent deproteinization, described in detail elsewhere [70] and allowing to measure acid labile and easily oxidizable compounds. Previously established ion pairing HPLC methods, in which the pairing reagent is represented by tetrabutylammonium hydroxide [70,71], were used in order to simultaneously separate high-energy phosphates (e.g., ATP), nicotinic coenzymes, GSH, MDA, and nitrite in the protein-free cell extracts (total injected volume of 200 µL). Separation was achieved through the use of a Hypersil C-18, 250 × 4.6 mm, 5 µm particle size column, provided with its own guard column, while the HPLC apparatus was composed by a SpectraSYSTEM P4000 pump system and a highly sensitive UV6000LP diode array detector, equipped with 5 cm light path flow cell. Identification and quantification of the compounds of interest in chromatographic runs of cell extracts were obtained by the comparison of retention times, absorption spectra, and area of the peaks (260 nm for high energy phosphates and nicotinic coenzymes, 266 nm for MDA, or 206 nm for GSH and nitrite) of chromatographic runs of mixtures containing known concentrations of true ultrapure standard mixtures.

### 4.7. Statistical Analysis

Statistical analysis was performed by using the 8.0 version of Graphpad Prism software (Graphpad software, San Diego, CA, USA). Student’s *t*-test was used to assess the statistical differences between two experimental groups. Only *p*-values of less than 0.05 were considered statistically significant. Data are always reported as the mean ± SD of at least 3 independent experiments.

## 5. Conclusions

In the present study, we were able to show that the incorporation of carbon-based nanoparticles (ECNs) into an artificial LS significantly decreased the biochemical alterations induced to alveolar (A549) and microglial (BV-2) cells by incubation with ECNs only. In particular, the presence of LS decreased oxidative/nitrosative stress (lower levels of NO, total ROS, and MDA), ameliorated antioxidant defenses (diminution of GSH depletion), and improved mitochondrial-related energy metabolism (increase of ATP and mitochondrial phosphorylating capacity). Even though further evidence is needed to evaluate the ability of LS to positively modulate the ECNs-induced biochemical alterations of target cells, the results generated in the present study make the base for the investigation of the ECNs + LS combination in vivo. A much better comprehension of the molecular and biochemical changes deriving from the interaction of “free” ECNs or ECNs + LS with living cells is of utmost importance before these highly promising tools might find the correct applications in the biomedical field.

## Data Availability

The data presented in this study are available on request from the corresponding author.
